# Neuroinflammation and Senescence Are Detected in Brainstems of Mice Latently Infected with HSV-1

**DOI:** 10.3390/pathogens15050510

**Published:** 2026-05-09

**Authors:** Melanie A. Whitmore, Kelly S. Harrison, Hafez Sadeghi, Bhuvana Plakkot, UdayKiran Venugopal, Chenoa Turtle, Madhan Subramanian, Clinton Jones

**Affiliations:** 1 Department of Veterinary Pathobiology, College of Veterinary Medicine, Oklahoma State University, Stillwater, OK 74078, USA; melanie.whitmore@okstate.edu (M.A.W.); hkellys@okstate.edu (K.S.H.); hafez.sadeghi@okstate.edu (H.S.); 2 Department of Physiological Sciences, College of Veterinary Medicine, Oklahoma State University, Stillwater, OK 74078, USA; bhuvana.plakkot@okstate.edu (B.P.); udaykiran.venugopal@okstate.edu (U.V.); chenoa.turtle@okstate.edu (C.T.)

**Keywords:** herpes simplex virus type 1 (HSV-1), latency, brainstem, locus coeruleus (LC), principal sensory nucleus of the spinal trigeminal tract (Pr5), neuroinflammation

## Abstract

Following acute infection, herpes simplex virus type 1 (HSV-1) establishes life-long latency in neurons. Although sensory neurons in trigeminal ganglia (TG) are primary sites for latency, the brainstem is also an important site for latency. The rationale for examining the principal sensory nucleus of the spinal trigeminal tract (Pr5) receives afferent inputs from TG. Notably, the (LC) is indirectly linked to Pr5. Our previous studies revealed that senescent cells and inflammation were detected in the Pr5 and LC of aged mice and young mice that are latently infected with HSV-1. To expand our understanding of how HSV-1 influences senescence and inflammation in Pr5 and LC, NanoString studies in mice latently infected with wild-type HSV-1 or a latency-associated transcript (LAT) null mutant (dLAT2903) was compared to age-matched uninfected C57Bl/6 male and female mice. LAT is the only viral gene abundantly expressed during latency, suggesting it influences cellular gene expression during latency. Cellular genes that regulate neuron differentiation, axonal projection, and pro-inflammatory mediators were more prevalent in mice latently infected with wild-type (wt) HSV-1 and dLAT2903 versus uninfected mice. Finally, these studies revealed that latency in Pr5 and LC is a dynamic process.

## 1. Introduction

Following acute infection, herpes simplex virus type 1 (HSV-1) establishes life-long latency in neurons within trigeminal ganglia (TG) and central nervous system [1,2], including the brainstem [3,4,5]. Following a burst of viral lytic cycle gene expression, viral gene expression is impaired, a subset of infected neurons survive, and latency is established and maintained. The latency-associated transcript (LAT), a complex locus that is the only viral gene abundantly expressed during latency, is reviewed in [1,2]. LAT expresses at least six micro-RNAs, two small non-coding RNAs, a stable intron, and three small transcripts that are antisense to the LAT. It is not clear whether a functional protein is expressed by the LAT. LAT products impair apoptosis [6], interfere with viral replication [1,2], and promote neuronal differentiation [7,8].

HSV-mediated encephalitis (HSE), the most prevalent viral encephalitis, occurs in 2000–4000 cases/year in the USA [9]. Despite antiviral therapies, mortality rates can be as high as 70% [10,11,12,13,14]. HSE leads to necrotic cell death due to virus replication and inflammation [15], which costs the USA approximately $2 billion/year [16]. Long-term complications occur despite antiviral treatment because approximately 2/3 of HSE cases are due to reactivation from latency [17]. HSE cases have also been reported in the brainstem [18,19], and generally, HSE cases in brainstem are also the result of reactivation from latency [3,4,5].

**Figure 1:**
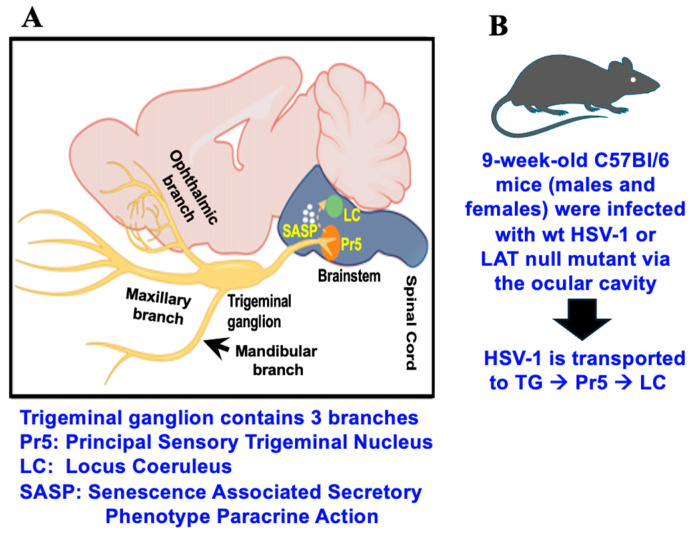
Schematic of mouse, location of mouse brain, TG, and brainstem. (**A**): Sagittal section of brain and location of LC and Pr5. (**B**): Schematic of infecting 9-week-old C57Bl/6 mice (males and females) with HSV-1. Mice were infected by instilling ocular mucosal surfaces with 100,000 pfu wt McKrae or dLAT2903 in 2–3 ul MEM. Eyelids were then closed and gently rubbed to facilitate virus infection. As denoted in Figure 1A, ocular HSV-1 will drain down the nasolacrimal duct. Consequently, virus is present in the nasal and oral cavity. Although infectious HSV-1 primarily enters TG via the ophthalmic brand, virus may also enter TG via the maxillary and mandibular branches.

Recent studies examined virus–host interactions in brainstem regions that receive afferent input from TG [20]. For example, the principal sensory nucleus of the spinal trigeminal tract (Pr5) receives afferent inputs from TG (Figure 1A). Pr5 contains many motor neurons that receive information about discriminative sensation, touch of the face, and conscious proprioception of the jaw [21]. Synaptic connections exist between the spinal trigeminal tract, which contains Pr5 [22], and locus coeruleus (LC). Hence, LC synaptic projections are indirectly linked to TG. LC projections reach all parts of the brain except the basal ganglia [23]. LC primarily contains medium-sized neurons with melanin granules [24] and is the principal site for norepinephrine synthesis in the brain. Threatening or stressful stimuli trigger norepinephrine release from LC; consequently, adrenal production of cortisol occurs. Notably, these novel LC functions are predicted to mediate HSV-1 replication. For example, key immediate early viral promoters, HSV-1 replication in cultured cells, and reactivation from latency are stimulated by the synthetic corticosteroid dexamethasone, which mimics the effects of stress, reviewed in [25].

Cellular damage in the nervous system can initiate senescence, a stress response characterized by irreversible cell cycle arrest and a distinct senescence-associated secretory phenotype (SASP). Senescence induces the expression of two cyclin-dependent kinase inhibitors such as p16 and p21, and pro-inflammatory cytokines and chemokines, reviewed in [26,27]. Senescent cells accumulate in the brain during aging and drive neurodegenerative processes. Notably, enhanced senescence and the expression of key inflammatory markers, including NLRP3 (NOD-, LRR- and pyrin domain-containing protein 3) levels are higher in the LC of female mice latently infected with an LAT null mutant (dLAT2903) versus wt McKrae HSV-1 when compared to age-matched male mice [28]. These differences were not observed in the Pr5 of female mice, suggesting that novel virus–host interactions occur in LC versus Pr5. NLRP3 activation is induced by virus infection and other cellular stressors [29], which can lead to pyroptosis, an inflammatory form of programmed cell death [30,31].

The goal of this study was to compare how HSV-1, wt versus dLAT2903, regulate pro-inflammatory gene expression in the Pr5 and LC of males and females of young (9-week old C57Bl/6) mice. NanoString studies were performed to confirm and expand our knowledge of how HSV-1 and LAT mediate gene expression in the LC and Pr5 of latently infected mice. These studies revealed there are significantly higher levels of pro-inflammatory mediators and immunological responses in the Pr5 and LC of latently infected mice versus age-matched control mice. Certain differentially expressed cellular genes exhibited sex-specific effects and LAT-specific effects in Pr5 versus LC during latency.

## 2. Materials and Methods

### 2.1. Viruses Used for This Study

The HSV-1 strains McKrae dLAT2903R (wt; LAT^+/+^) and dLAT2903 (LAT^-/-^) were provided by the late Dr. Steven Wechsler. dLAT2903R virus was rescued from dLAT2903 by restoring LAT sequences through homologous recombination. This rescued virus exhibits growth characteristics in mice and rabbits that are comparable to those of the parental wt HSV-1 McKrae strain [32,33], and is therefore denoted as wt HSV-1 in this study.

Monkey kidney (Vero) cells were grown as monolayers in minimal essential medium (MEM) supplemented with 10% fetal bovine serum (FBS), 2 mM L-glutamine, 100 IU/mL penicillin, 100 µg/mL Streptomycin at 37 °C and 5% CO_2_. Virus stocks were prepared by infecting Vero cells at a low MOI (0.01 pfu/cell). Cells were harvested once at least 80% displayed cytopathic effects. To release the virus, cells underwent three freeze–thaw cycles, followed by centrifugation to remove cellular debris. The supernatant containing cell-free virus was aliquoted and stored at (−80 °C). The virus was tittered on Vero cell monolayers to calculate the PFU/mL of each stock prior to infection of mice.

### 2.2. Mouse Studies

All mice were housed and handled in agreement with the Oklahoma State University Institutional Animal Care and Use Committee, approved animal use protocols; IACUC-24-58, approved on 17 December 2024 and IACUC-21-53, approved on 13 December 2024. Eight-week-old male and female C57BL/6J mice were obtained from Jackson Laboratories and allowed to acclimate to laboratory conditions (12 h light/dark cycles, 5 animals/cage) for 7 days prior to infection. For the studies discussed below, groups included five females and five males infected with wt HSV-1 or dLAT2903, along with five mice of each sex that were mock-infected. Mice were anesthetized using standard isoflurane/oxygen vaporization and then infected with ~2 × 10^5^ PFU of dLAT2903R or dLAT2903 in 2–3 µL MEM per eye. The virus was applied to the ocular cavity, after which the eyelid was closed and gently rubbed to facilitate HSV-1 infection (Figure 1B) [34]. Ocular scarification was not performed, as the McKrae strain can infect mice without this procedure.

Following infection, mice were observed for signs of HSV-1-induced encephalitis (HSE), including tremors, forehead swelling, or weight loss. Animals exhibiting these symptoms were euthanized using isoflurane overdose and then cervical dislocation. C57BL/6J mice are highly resistant to the effects of HSV-1 [35,36] and ~25% of the mice exhibited HSE symptoms. Surviving mice often show symptoms of acute ocular herpes infection, such as ocular redness, discharge, and/or fur loss near the inoculation site. During acute infection, symptomatic mice were treated twice a day with Neomycin/polymyxin B/Bacitracin zinc ophthalmic ointment (Bausch and Lomb, Tampa, FL, USA) to prevent secondary bacterial infections. Mice were considered latently infected by 30 days post-infection. Hence, mice were euthanized and brainstem samples were collected from 30 to 34 days after infection.

### 2.3. Microdissection of Brainstem

Serial coronal sections (60 µm thick) of brainstem were obtained using a cryostat (Leica CM3050S, Leica Biosystems, Wetzlar, Germany), and the sections were placed on super frost plus slides. Pr5 and LC were micro dissected using the Palkovit’s punch technique and a mouse brain stereotaxic atlas as a reference [21,37]. Bilateral micro punches were collected from 60 µm thick coronal sections based on stereotaxic coordinates. Punches collected from Pr5 and LC were preserved in RNA*later* (ThermoFisher, Invitrogen, Carlsbad, CA, USA, Cat #AM 7020, Waltham, MA, USA) at −20 °C until shipped.

### 2.4. NanoString nCounter Analysis

Total RNA prepared from the Pr5 and LC was hybridized to a mouse neuroinflammation panel (NanoString Technologies, Seattle, WA, USA, Catalog #XT-CSO-MNROI1-12) of target-specific fluorescent barcodes. The panel contains 770 genes, including 13 internal reference genes for data normalization. Subsequent quality control, normalization, and between-group comparisons for differential gene expression were done using the nSolver Analysis Software from NanoString Technologies. The analysis was performed by Canopy Biosciences (St. Louis, MO, USA) and we performed the bioinformatic studies. The Database for Annotation, Visualization, and Integrated Discovery (DAVID) was used for functional enrichment analysis [38,39], and results with a *P*-value ≤ 0.05 were considered statistically significant. The data from the NanoString studies was deposited in the NCBI GEO depository under the accession number GSE326523.

## 3. Results

### 3.1. Gene Expression Profile in the Pr5 of Latently Infected Mice

**Figure 2:**
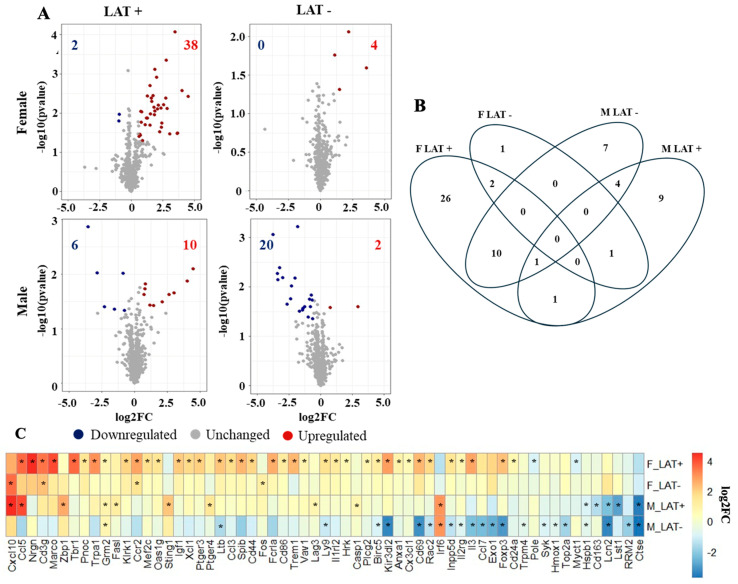
Overview of differential gene expression in Pr5. (**A**) Volcano plots of differentially expressed genes (DEGs) comparing mice latently infected (32 days after infection) with wt HSV-1 and dLAT2903 in males and females to uninfected mice of the same sex. (**B**) Venn diagram of DEGs comparing wt HSV-1 versus dLAT2903 in male (M) and female (F) mice and compared to age-matched uninfected mice. (**C**) Heatmap of DEGs as described for B. The group in which a gene was significant is marked by an asterisk. DEGs had a *P*-value < 0.05 and fold change > |1.5|.

NanoString RNA sequencing assessed neuroinflammation in the Pr5 and LC of mice latently infected with wt HSV-1 or the LAT^-/-^ mutant (dLAT2903) (Figure 2B). The nSolver analysis revealed 40 differentially expressed genes (DEGs) in the Pr5 of females latently infected with wt HSV-1 compared to uninfected females, whereas only four DEGs were found in females latently infected with dLAT2903 (Figure 2A). Neurogranin (Nrgn) RNA levels were upregulated 23-fold in females infected with wt HSV-1. The Nrgn gene encodes a protein enriched in postsynaptic compartments that binds calmodulin and: (1) enhances synaptic plasticity, (2) is associated with the decline of memory in elderly people independent of Alzheimer disease markers [40], (3) is reduced in prefrontal cortex regions in schizophrenia patients [41], (4) is a biomarker in mild traumatic brain injury [42], and (5) its activity is mediated by protein kinase C phosphorylation (Table 1) [43]. The pro-inflammatory chemokine, C-C motif chemokine ligand 5 (Ccl5) was expressed ~13-fold higher in female mice latently with wt HSV-1, whereas the pro-inflammatory cytokine Cxcl10 was expressed ~12 fold higher in Pr5 of females latently infected with dLAT2903. Ccl5 also recruits a superfamily of cytokines, interleukins, interferons, and chemokines that facilitate inflammatory responses, chronic diseases, and cancer, reviewed in [44]. 

Sixteen genes were differentially expressed in males latently infected with wt HSV-1 including Cxcl10, which was upregulated ~22-fold (Figure 2A). Cxcl10 attracts cells to sites of infection and inflammation [45]. Most of the other twenty-two DEGs identified in the Pr5 of the male LAT-group were downregulated (Figure 2A). Cathepsin E (Ctse), which encodes a protease involved in antigen presentation [46], was the highest suppressed gene in males latently infected with dLAT2903 (Table 1). Two genes were differentially upregulated in females latently infected with wt or dLAT2903 versus uninfected mice, CD3 gamma subunit (Cd3g) and C-C Motif chemokine receptor 2 (Ccr2) (Figure 2C). Ccr2 encodes a G-protein-coupled chemo-receptor that increases monocyte infiltration in response to the chemokine CCL2. Ccr2 also possesses functions independent of G-protein-coupled chemokine receptor functions, including regulating extracellular chemokine availability during normal immunological responses or inflammatory conditions [47].

**Figure 3:**
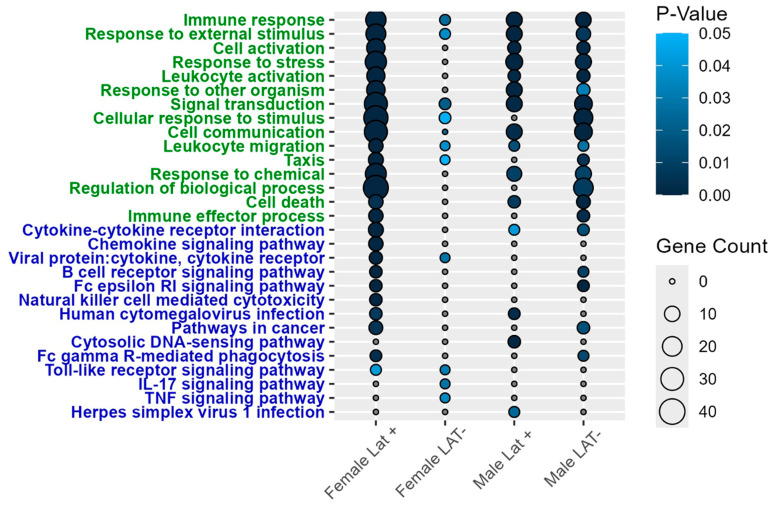
Gene ontology (green) and KEGG (blue) pathway enrichment analysis of DEGs in the Pr5, with *P*-value < 0.05 considered significant.

In the Pr5 of males, glutamate metabotropic receptor 2 (Grm2), interferon regulatory factor 6 (Irf6), lipocalin-2 (Lcn-2), and Ctse were differentially expressed regardless of whether they were latently infected with wt McKrae or dLAT2903 (Figure 2B). Lcn-2 is linked to increased neurodegeneration [48] and expression is induced in the CNS of mice infected with West Nile Virus that led to encephalitis [49]. The only DEG shared by males and females infected with wt HSV-1 was Ccl5 (Figure 2C and Table 1). To better understand functions associated with genes differentially enriched in the Pr5 of mice latently infected with HSV-1, the DAVID platform was used for a pathway enrichment analysis. Immune responses were among the top three gene ontology (GO) terms across all four treatments, including 22 DEGs in females and 11 in males latently infected with wt HSV-1 (Figure 3). Similarly, the most prevalent KEGG pathway was cytokine–cytokine receptor interaction.

Changes in cell death and HSV-1 infection were also detected in Pr5 when latently infected mice were compared to uninfected controls (Figure 3). Interleukin 3 (IL-3) was the most upregulated gene out of eight DEG listed for cell death in females latently infected with wt HSV-1. While the loss of IL-3 is associated with cell death, its expression is also neuroprotective and promotes antiviral immune responses [50]. Genes that mediate cell death were not detected in females latently infected with dLAT2903. Z-DNA binding protein 1 (Zbp1) had the greatest change in gene expression among DEG (~6-fold increase) that regulates cell death in males latently infected with wt HSV-1. ZBP1 is a sensor for double-stranded DNA and RNA helices that are in the unusual left-handed Z conformation, which is referred to as Z-DNA and Z-RNA. ZBP1 interactions with double-stranded Z-DNA or Z-RNA induce type I interferon and programmed cell death (apoptosis, necroptosis, and pyroptosis), and activate inflammatory signaling via NF-kB, reviewed in [51,52]. The most downregulated gene in the cell death pathway for males latently infected with wt HSV-1 or dLAT2903 was Lcn-2, in male mice latently infected with WT HSV-1, Ccl5, Fas ligand (Fasl), and stimulator of interferon response cGAMP interactor 1 (Sting1). STING is a cytosolic protein that binds DNA in the cytosol; consequently, interferon pathways and apoptosis, pyroptosis, and/or necroptosis can be activated, reviewed in [53,54].

### 3.2. Gene Expression Profile in the LC of Latently Infected Mice

**Figure 4:**
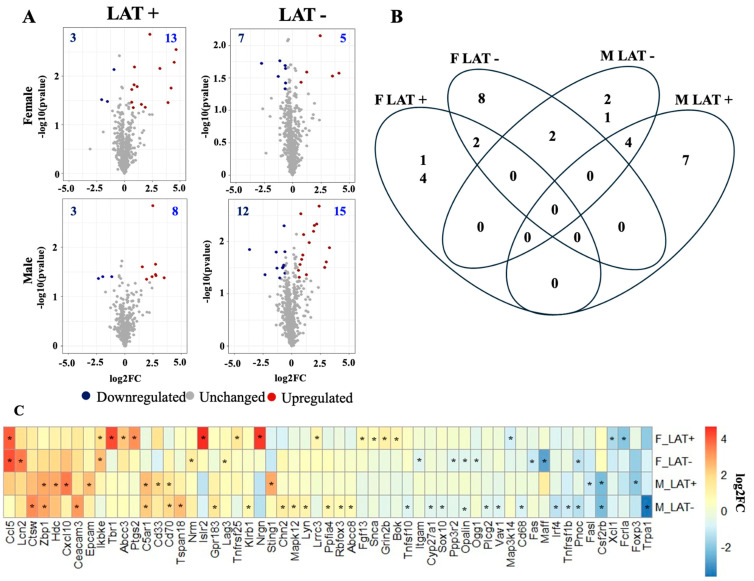
Overview of differential gene expression in LC. (**A**) Volcano plots of differentially expressed genes (DEGs) comparing mice latently infected with wild-type (LAT+) or dLAT2903 (LAT-) HSV-1 (34 days after infection) to uninfected mice of the same sex. (**B**) Venn diagram of DEGs among all four comparisons. (**C**) Heatmap of genes differentially expressed in at least one comparison. The group in which a gene was significant is marked by an asterisk. DEGs had a *P*-value < 0.05 and fold change > |1.5|.

The LC of females latently infected with wt HSV-1 contained 16 DEGs (Figure 4A). For example, the immunoglobulin superfamily containing leucine-rich repeat 2 (Islr2) showed a 25-fold increase in females latently infected with wt McKrae (Table 1). Islr2, also known as Linx, encodes a protein that promotes axon extension, whereas axonal growth is stunted in mice that do not express Islr2 [55,56]. Furthermore, Islr2 interacts with the Rho-kinase, which is important for increasing neuronal cell body area and neurite extension. Three more of the top genes in females latently infected with wt HSV-1 were identified in Pr5 and LC: Nrgn, Ccl5, and T-box brain gene 1 (Tbr1). Like Islr2 functions, Tbr1 is a transcription factor that promotes neuron differentiation and axonal projection [57,58]. In females latently infected with dLAT2903, 12 DEGs were identified. Of these 12 genes, Ccl5 had the greatest change in gene expression, which was approximately a 17-fold increase.

Males contained 11 and 27 DEGs when latently infected with wt HSV-1 or dLAT2903, respectively (Figure 4A). The magnitude of transcriptional changes was less in LC of males than in females. For example, the innate immune gene stimulator of interferon response cGAMP interactor 1 (Sting1) was the highest DEG in LAT+ males, with a 7-fold increase when compared to age-matched uninfected males (Table 1). The transient receptor potential ankyrin 1 (Trpa1), a cellular stress and inflammatory response sensor, was the only gene in LAT- males that had an expression level changed by more than 10-fold when compared to uninfected mice (Table 1) [59].

Only two genes were differentially expressed among all LAT- mice in the LC, oligodendrocytic myelin paranodal inner loop protein (Opalin) and prepronociceptin (Pnoc), while the LAT+ groups did not have any common DEGs (Figure 4C). In addition to Ccl5, an antiviral gene, an inhibitor of nuclear factor kappa B kinase subunit epsilon (Ikbke), was elevated in the LC of all latently infected females. Male mice contained four DEGs in common (latently infected with wt HSV-1 and dLAT2903); Z-DNA binding protein 1 (Zbp1), complement C5a receptor 1 (C5ar1), and Cd70 were upregulated, but the colony-stimulating factor 2 receptor subunit beta (Csf2rb) was downregulated (Figure 4C). Interestingly, RNA levels of the TRPA1 ion channels were reduced in the LC of males latently infected with dLAT2903 (Table 1), whereas it was not differentially expressed in the LC of females latently infected with wt HSV-1 or dLAT2903. TRPA1 expression has many functions, including triggering pain signals, inflammation and apoptosis, reviewed in [60].

**Figure 5:**
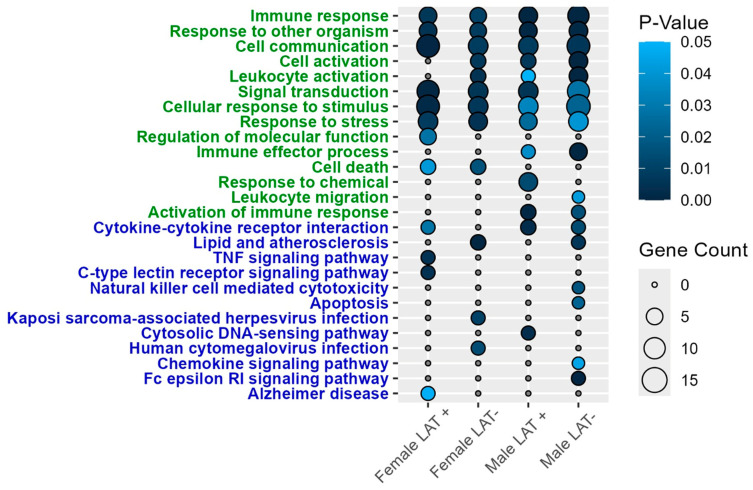
Gene ontology (green) and KEGG (blue) pathway enrichment analysis of DEGs in the LC, with *P*-value < 0.05 considered significant.

The DAVID analysis revealed that many of the same GO terms are enriched in the LC as in the Pr5. These are highlighted by the immune response, which included five to 10 DEGs across all four comparisons (Figure 5). However, the majority of the KEGG pathways were unique in LC versus Pr5. The most statistically significant KEGG pathway for females latently infected with wt HSV-1 was TNF signaling, while lipid and atherosclerosis were significantly different in female mice latently infected with dLAT2903. In males, cytosolic DNA-sensing was the top prediction for the LAT+ group, whereas Fc epsilon RI signaling was the highest DEG in the LAT- group. Interestingly, Fc epsilon RI RNA is also detected in superior cervical ganglion (SCG) [61] and small TG neurons [62]. Furthermore, Fc epsilon RI can be activated by antigen and transmits a signal along nerve fibers in vitro and in vivo. Thus, increased Fc epsilon RI expression in neurons may trigger neurogenic inflammation.

### 3.3. Expression of Immune Mediators in the Brainstem During HSV-1 Latency

**Figure 6:**
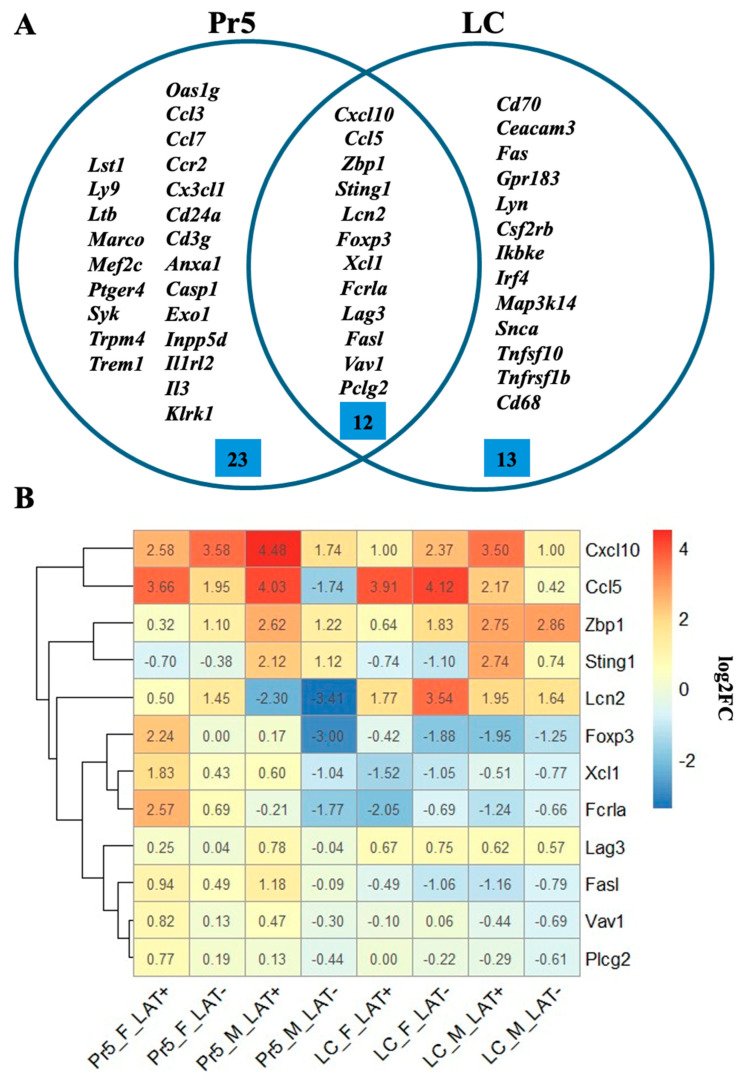
DEGs associated with immune responses. (**A**): Venn diagram of DEGs associated with immune response in the Pr5 and LC. (**B**): Heatmap of immune response genes found in both the Pr5 and LC. DEGs had a *P*-value < 0.05 and fold change > 1.5.

Immune response was the most prevalent pathway detected by enrichment analysis in the brainstem of mice latently infected with HSV-1, including 48 DEGs in at least one of the eight comparisons (Figure 6A). Of these, 12 genes were detected in both Pr5 and LC (Figure 6A). For example, Cxcl10 and Ccl5 were upregulated in latently infected mice (Figure 6B) and these genes are important for T-cell recruitment (Table 2). Four additional genes differentially regulated in the brainstem possessed roles in the differentiation or activation of T-cells, namely Foxp3 [63], [Xcl1], Lag3 [64], and Vav1 [65] (Table 2).

Notably, Lipocalin-2 (Lcn-2) was expressed more than 8-fold higher in the LC of females latently infected with dLAT903, which was slightly higher than wt HSV-1. Lcn-2 was increased less than 4-fold in males or females latently infected with wt HSV-1. In Pr5, Lcn-2 expression was reduced in mice latently infected with wt HSV-1 or dLAT2903. Lcn-2 is produced in activated astrocytes following inflammatory stress, and is subsequently secreted, which leads to damaged neurons, neurodegeneration, and neuronal loss, reviewed in [48]. Lcn-2 also binds to the Lcn-2 receptor (24p3R) in astrocytes leading to increased activation in astrocyte and microglia. Interestingly, the Z-DNA binding protein 1 (Zbp1) and stimulator of interferon genes (Sting1) expression were more pronounced in males versus females (uninfected versus latently infected mice (Figure 6B). As denoted above, Zbp1 and Sting1 proteins trigger inflammation (Table 2).

## 4. Discussion

This study revealed that Pr5 and LC expressed numerous cellular genes that promote inflammation, neurodegeneration, and immune responses in latently infected mice, but not age-matched uninfected mice, regardless of sex. These findings imply that increased inflammation and immune responses were, in part, due to low levels of sporadic viral gene expression in Pr5 and LC in latently infected mice. Support for this premise comes from studies that concluded low levels of lytic cycle viral gene expression occur due to the cell-intrinsic transcriptional responses in TG of latently infected mice [71]. This observation was confirmed by an independent study that these events are denoted spontaneous molecular reactivation [72]. Another independent study concluded that a lytic viral protein regulates latent viral chromatin structure during latency in TG [73]. Collectively, these studies imply that low levels of lytic cycle viral gene expression occur in Pr5 and LC in latently infected mice. Additional studies are necessary to determine if certain viral genes are expressed in the Pr5 and LC of latently infected mice or if random viral genes are sporadically expressed at low levels and whether reactivation from latency occurs. This could be difficult because Pr5 and LC are much smaller than TG. Furthermore, there is at least 10 times lower levels of viral DNA in the Pr5 and LC of mice latently infected with wt HSV-1 when compared to the TG of latently infected mice [28].

Notably, DEGs that enhance neuronal functions were identified in the Pr5 or LC of mice latently infected with wt HSV-1. For example, Nrgn expression was increased 23-fold in the Pr5 of female mice latently infected with wt HSV-1 when compared to mice latently infected with dLAT2903. The Nrgn protein is enriched in postsynaptic compartments, which enhances synaptic plasticity [40], but is reduced in prefrontal cortex regions in schizophrenia patients [41]. Furthermore, Islr2 was expressed at a 25-fold increase in females latently infected with wt McKrae versus dLAT2903 or age-matched uninfected female mice. The Islr2 protein promotes axon extension and axonal growth is reduced in knockout Islr2 mice [55,56]. Islr2 also interacts with the Rho-kinase, which increases neuronal cell body area and neurite extension. Thirdly, the transcription factor Tbr1 regulates neural stem cell differentiation and impairs astrocyte formation primarily in the olfactory bulb and was in the top 5 of DEGs in female LC and Pr5 latently infected with wt HSV-1 [57,58]. Interestingly, genes that promote neuronal well-being were generally increased in mice latently infected with wt HSV-1, but not dLAT2903. This trend implies that LAT expression directly or indirectly promotes the expression of these important cellular genes.

In certain circumstances, the absence of LAT expression correlated with distinct differentially expressed cellular genes in the Pr5 or LC of mice latently infected with dLAT2903 versus wt HSV-1. For example, the neurotoxin (Lcn-2) was expressed at higher levels in LC of females latently infected with wt HSV-1 or dLAT903 but not Pr5, regardless of which virus was used for infection. Furthermore, RNA levels of the TRPA1 ion channels were reduced in the LC of males latently infected with dLAT2903, whereas it was not differentially expressed in the LC of females latently infected with wt HSV-1 or dLAT2903. Cxcl10 expression was significantly increased in the Pr5 of females latently infected with dLAT2903 but was only expressed in the Pr5 of male mice latently infected with wt HSV-1. The Ccl5 pro-inflammatory cytokine was one of the few genes significantly increased in the Pr5 and LC of mice latently infected with wt HSV-1 or dLAT2903. The increase in pro-inflammatory cytokines is predicted to play a role in maintaining lymphocytes that infiltrate the TG and brainstem in latently infected mice and humans, which reduces the frequency of reactivation from latency.

The first 1.5 kb of LAT coding sequences is necessary for inhibiting apoptosis in transient transfection, which correlates with efficient reactivation from latency [6,74]. LAT-expressing mouse neuroblastoma cells (C1300) maintain protein kinase B (Akt) expression and phosphorylation when cells are subjected to cold shock, which induces apoptosis [8]. C1300 control cells, which do not express LAT, contain reduced Akt protein levels plus phosphorylation and cell death after cold shock. It is well established that Akt impairs apoptosis and enhances axon growth in neurons [75], and the Akt/GSK-3beta/beta-catenin signaling axis is a positive regulator of neuronal survival [76]. Thus, it was surprising that significant differences were present in the Akt/GSK-3beta/beta-catenin signaling axis in the PR5 or LC of uninfected controls, which was not different in mice latently infected with wt HSV-1 or dLAT2903.

Considering Pr5 and LC contain distinct types of neurons that have very distinct functions, it was not surprising that the latent infection of these two brainstem regions gave rise to different cellular genes that were differentially expressed. Notably, LC, a nucleus in the pons, plays pivotal roles in stress and anxiety [77,78]. Furthermore, C57Bl/6J female mice exhibit stronger anxiety-relative behaviors than males. In humans, damage to LC is an early indicator of Alzheimer’s disease (AD) [79,80,81] and women have a 2-fold increase in AD incidence [82,83].

Certain differentially expressed genes did not appear to play a direct role in neuroinflammation, neuronal differentiation, and/or neuronal health in the Pr5 and LC of latently infected mice. For example, C5a is a component of the complement that binds and activates two distinct receptors (C5aR1 and C5aR2), reviewed in [84]. C5aR1 is a G-protein-coupled receptor and C5a is a complement component. These interactions can lead to increased inflammatory responses, the expression of adhesion molecules, and vascular permeability. C5a also attracts phagocytic cells to sites of infection or recruitment of antigen-processing cells. CD70 is a co-stimulator of T-cell and B-cells that improve activation, proliferation and survival: consequently, CD70 enhances immune response in B-cells, T-cells, NK cells, and mature dendritic cells, reviewed in [27]. CD70 expression in T and B-cells is also stimulated by inducing the expression of T- and B-cell receptors. CD70 expression in dendritic cells is induced via Toll-like receptors, which triggers CD40 ligation. CD70 is also induced on NK cells via IL-15 stimulation. Interactions between CD27 and CD70 recruit adaptor proteins, TRAF2 and TRAF5: consequently, the NF-kB and c-Jun N-terminal kinase (JNK) pathway is activated. Based on the functions of C5a and CD70, we predict these gene products impair reactivation from latency by mediating inflammatory and immune responses.

CSF2RB (colony-stimulating factor 2 receptor subunit beta) is a cell surface receptor that regulates certain immune responses, reviewed in [85]. Notably, CSF2RB forms heterodimers with several important immune regulators: interleukin-3 receptor subunit alpha, interleukin-3 receptor subunit beta, and colony-stimulating factor 2 receptor subunit alpha. Consequently, these novel heterodimers bind distinct receptors and induce expression of different cytokines, including interleukin-3 (IL-3), interleukin-5 (IL-5), and granulocyte–macrophage colony-stimulating factor (GM-CSF). CSF2RB also constitutively interacts with and activates JAK1, which initiates the JAK-STAT pathway. Notably, CSF2RB expression in bladder and esophageal cancers generally leads to an unfavorable prognosis. Conversely, CSF2RB expression correlates with a favorable prognosis in cervical squamous cell carcinoma, certain breast cancers, and endo-cervical adenocarcinoma. Like many of the differentially expressed genes in Pr5 and LC of latently infected mice, it appears CSF2RB impairs lytic cycle viral gene expression, which may lead to programmed cell death and/or reactivation from latency.

Infectious agents, including HSV-1 and Varicella Zoster Virus, are predicted to increase neurodegeneration in certain AD cases [86,87]. This topic is controversial, and it is unlikely that HSV-1 is the primary AD cofactor. However, HSV-1 may serve as a cofactor in certain people. For example, females have a 1 in 5 chance of developing AD; however, only 1 in 11 men develop AD. Ccl5 expression was higher in the LC of females latently infected with dLAT2903 or wt HSV-1 relative to LC derived from males. Although Lcn-2 expression was higher in the LC of female mice latently infected with dLAT2903, it was reduced in the Pr5 of male mice infected with either dLAT2903 or wt HSV1 and did not show a dramatic increase in the Pr5 of females. Morphological changes have been identified in the brainstem of patients, in particular LC, that develop AD [88,89]. These studies imply that harboring life-long HSV-1 latency can play a role in neurodegeneration and/or alter other functions in LC neurons. Finally, it is also possible that neurons in other regions in the brainstem are influenced by HSV-1 latency and reactivation from latency.

## 5. Conclusions

This study supports the concept that HSV-1 influences certain cellular genes and signaling pathways during latency. The mechanism by which HSV-1 alters the expression of certain cellular genes is not understood. However, HSV-1 latency induces the expression of genes that regulate immune responses and neuroinflammation, which we predict reduces reactivation from latency. The finding that latency also induces the expression of certain cellular genes that maintain normal neuronal processes, which promotes life-long latent infections and is predicted to benefit the host. In closing, this study revealed that the maintenance of latency in Pr5 and LC is not a quiescent process and in certain cases we suggest neurodegeneration can occur.

## Figures and Tables

**Table 1:** The five DEGs from each comparison with the greatest change in gene expression during latency versus uninfected mice.

Pr5				LC		
Symbol	FC	*P*-Value		Symbol	FC	*P*-Value
**Female LAT+**				**Female LAT+**		
*Nrgn*	22.63	0.004		*Islr2*	24.93	0.003
*Marco*	16.34	0.003		*Nrgn*	22.01	0.005
*Ccl5*	12.64	0.033		*Tbr1*	18.38	0.018
*Tbr1*	12.47	0.033		*Ccl5*	15.03	0.035
*Cd3g*	8.22	0.000		*Ptgs2*	9.06	0.007
**Female LAT** **-**				**Female LAT** **-**		
*Cxcl10*	11.96	0.026		*Ccl5*	17.39	0.027
*Cd3g*	4.59	0.009		*Lcn-2*	11.63	0.030
*Ccr2*	2.81	0.049		*Ikbke*	5.50	0.007
*Fos*	2.17	0.017		*Pnoc*	−2.43	0.030
-----------------------------------------------				*Maff*	−6.73	0.019
**Male LAT+**				**Male LAT+**		
*Cxcl10*	22.32	0.008		*Sting1*	6.68	0.022
*Ccl5*	16.34	0.013		*C5ar1*	5.58	0.001
*Irf6*	8	0.022		*Cd70*	2.95	0.025
*Lst1*	−7.16	0.010		*Foxp3*	−3.86	0.040
*Ctse*	−11.71	0.001		*Csf2rb*	−4.99	0.043
**Male LAT** **-**				**Male LAT** **-**		
*Foxp3*	−8	0.007		*C5ar1*	4.56	0.005
*Cd69*	−9.32	0.004		*Cd70*	3.94	0.005
*Kir3dl2*	−10.34	0.007		*Pnoc*	−2.58	0.016
*Lcn-2*	−10.63	0.005		*Csf2rb*	−4.99	0.043
*Ctse*	−13.36	0.001		*Trpa1*	−12.64	0.014

**Table 2:** Immune response genes differentially enriched in the Pr5 and LC.

Gene	Function
**Cxcl10**	Pro-inflammatory chemokine with diverse functions. Promotes monocyte, natural killer, and T-cell migration. Activates microglia. Protects against recurrent HSV-1 infection [66].
**Ccl5**	Chemokine that facilitates inflammatory responses. Induces adhesion and migration of leukocytes [44].
**Zbp1**	Binds to Z-form nucleic acids. Central regulator of PANoptosis [52].
**Sting1**	Mediates pro-inflammatory cytokine and type I interferon production, regulates autophagy [54].
**Lcn-2**	A neutrophil gelatinase-associated lipocalin. Bacteriostatic effect by sequestering iron-containing siderophores [49].
**Foxp3**	A transcription factor essential for regulatory T-cell development [67].
**Xcl1**	Highly expressed in activated CD8+ T-cells; chemoattractant for CD8+ DC cells [68].
**Fcrla**	Intracellular protein selectively expressed in B-cells. May act as a chaperone in the ER for IgA, IgM, IgG [63].
**Lag3**	Inhibitor of T-cell effector functions; can mediate T-cell exhaustion [64].
**Fasl**	Expressed primarily on activated T-cells and natural killer cells; Fas/Fasl signaling triggers apoptosis [69].
**Vav1**	Important in hematopoiesis; plays a role in T-cell and B-cell activation [65].
**Plcg2**	Transmembrane signaling enzyme that generates IP3 and DAG secondary messengers important for transmitting signals from growth factor receptors and immune system receptors [70].

## Data Availability

The data from the NanoString studies was deposited in NCBI GEO depository under the accession number GSE326523. This data is scheduled to be publicly available on 1 July 2026.
